# Increased Insular Cortical Thickness Associated With Symptom Severity in Male Youths With Internet Gaming Disorder: A Surface-Based Morphometric Study

**DOI:** 10.3389/fpsyt.2018.00099

**Published:** 2018-04-03

**Authors:** Shuai Wang, Jing Liu, Lin Tian, Limin Chen, Jun Wang, Qunfeng Tang, Fuquan Zhang, Zhenhe Zhou

**Affiliations:** ^1^Department of Psychiatry, The Affiliated Wuxi Mental Health Center of Nanjing Medical University, Wuxi, China; ^2^Wuxi Tongren International Rehabilitation Hospital, Wuxi, China; ^3^Wannan Medical College, Wuhu, China; ^4^Department of Substance Dependency, The Affiliated Wuxi Mental Health Center of Nanjing Medical University, Wuxi, China; ^5^Department of Medical Imaging, Wuxi People’s Hospital Affiliated to Nanjing Medical University, Wuxi, China

**Keywords:** cortical thickness, insula, Internet gaming disorder, surface-based morphometry, symptom severity

## Abstract

With the rising increase in Internet-usage, Internet gaming disorder (IGD) has gained massive attention worldwide. However, detailed cerebral morphological changes remain unclear in youths with IGD. In the current study, our aim was to investigate cortical morphology and further explore the relationship between the cortical morphology and symptom severity in male youths with IGD. Forty-eight male youths with IGD and 32 age- and education-matched normal controls received magnetic resonance imaging scans. We employed a recently proposed surface-based morphometric approach for the measurement of cortical thickness (CT). We found that youths with IGD showed increased CT in the bilateral insulae and the right inferior temporal gyrus. Moreover, significantly decreased CT were found in several brain areas in youths with IGD, including the bilateral banks of the superior temporal sulci, the right inferior parietal cortex, the right precuneus, the right precentral gyrus, and the left middle temporal gyrus. Additionally, youths with IGD demonstrated a significantly positive correlation between the left insular CT and symptom severity. Our data provide evidence for the finding of abnormal CT in distributed cerebral areas and support the notion that altered structural abnormalities observed in substance addiction are also manifested in IGD. Such information extends current knowledge about IGD-related brain reorganization and could help future efforts in identifying the role of insula in the disorder.

## Introduction

According to the authoritative announcement of China Internet Network Information Center, till December 2017, the population of netizens in China has reached 772 million, accounting for about one-fifth of the total population of Internet users worldwide.^1^ With the rapid popularity of the Internet, the phenomenon of clinical impairments or distress caused by maladaptive use of the Internet has grasped the attention of medical and public health professionals (1–7). Research on maladaptive use of the Internet has become a rapidly evolving field of study (8, 9). In acknowledgment of the studies that have already been published in this field, the Section III of DSM-5 classified “Internet gaming disorder” (IGD) as a condition in need of further research before being officially recognized as an independent clinical disorder (10). As a probable candidate for behavioral addiction, IGD was defined in particular as “persistent and recurrent use of the Internet to engage in games” (11) and has gained massive attention worldwide. Scholars within the field have been motivated to provide empirical evidence for this potential clinical category by applying different study approaches such as epidemiology, psychosociology, and neuroimaging. For example, epidemiological studies have shown that the overall prevalence of IGD ranged from 0.7 to 15.6% in studies of naturalistic populations, with an average percentage of 4.7% over the years (12). In addition, several theoretical models have been proposed for inspiring clear hypotheses on the mechanisms underlying the clinical phenomenon of IGD, which can be useful for the theory-driven development of assessment tools and treatments (12–15).

The technological advancement of neuroimaging, especially non-invasive magnetic resonance imaging (MRI), has made it possible to assess both anatomical and functional brain characteristics of IGD (9). Convergent evidence has indicated that brain structural alterations were associated with individuals with IGD, which suggested an underpinning neuroscientific basis for IGD (8). For example, Han et al. (16) reported increased gray matter volume of the left thalamus in individuals with IGD, and Zhou et al. reported decreased gray matter density of the left cingulate cortex and left insula in individuals with IGD (17). With regard to those structural alterations, there was an influential explanation that the neural mechanisms underlying IGD resemble those of substance addiction (14, 18). Although such behavioral addictions do not involve a chemical intoxicant or substance, study evidence revealed that many aspects of behavioral addiction are similar to those of substance addiction (19, 20). For example, a common neurobiological feature during the resting state (21) and similar impulsivity and executive dysfunctions have been reported between IGD and alcohol use disorder (20). An open question thus is whether these altered structural abnormalities observed in substance addiction also manifest in IGD.

In the past decades, tremendous progress has been made in the techniques and applications of cortical surface morphometry based on structural MRI (22). Previous studies have indicated that surface-based brain mapping may offer advantages over volume-based brain mapping to capture the fine structure of cortical anatomy, since it provides a series of cortical measures that possess anatomical meanings, such as cortical thickness (CT) (22, 23). To the best of our knowledge, so far, very few studies have conducted surface-based brain mapping in the individuals with IGD. Reassuringly, as comparable references, one study has demonstrated the reduction of orbitofrontal CT in male adolescents with Internet addiction (24), and the other revealed a changed CT pattern in late adolescence with online gaming addiction (25). However, both studies were conducted before the publishing of DSM-5, and different criteria were applied throughout those studies. It is our belief that the features of cortical anatomy in IGD are not well known; neither is its association with symptoms of IGD. Therefore, it is necessary to assess the morphological features of IGD using the new DSM-5 approach. In the present study, we used surface-based morphometry (SBM) approaches to examine CT changes of the whole brain in male youths with IGD. According to previous findings derived from studies on IGD (8, 24, 25) and substance addiction (26), we hypothesized that male youths with IGD may have increased CT in the insula. Considering that the insula has been proposed to be crucial for the formation and maintenance of IGD (15), we further speculated that increased insular CT may be associated with symptom severity in male youths with IGD.

## Materials and Methods

### Participants

All participants were recruited from local universities and the surrounding community *via* advertisements and word of mouth. Participants were then pre-selected through an online questionnaire and telephone screening. Given the higher prevalence of Internet addiction in males versus females in China (27, 28), only male participants were included. Forty-eight youths who reported Internet gaming as their primary online activity met at least five of the nine DSM-5 criteria for IGD (10). Participant’s Internet addictive behavior was assessed with a Chinese version of Internet Addiction Test (IAT) (29). IAT includes 20 items on a 5-point Likert scale (scored from 1 to 5) indicating the level of Internet usage, with good internal consistency and concurrent validity (30, 31). The higher the score, the greater the problems caused by Internet usage. All IGD subjects satisfied with their score on the IAT more than the proposed cutoff score (i.e., ≥50) (32, 33). Male youths who dissatisfied the proposed criteria for IGD were pre-selected as normal controls (NCs). Among them, 32 participants were determined as NCs based on their score of less than 30 on the IAT. NCs satisfied with fewer than four of the nine criteria for IGD proposed by DSM-5. All participants were right-handed as assessed with the Edinburgh Handedness Inventory (34). A brief structured clinical interview tool, the Mini International Neuropsychiatric Interview (35), was used to screen for several psychiatric disorders. Exclusion criteria for the participants included intracranial pathology, brain injury, neurological disorder, several psychiatric disorders, substance abuse, contraindications for MRI examinations, and excessive head motion. The demographic characteristics of youths with IGD and NCs are summarized in Table 1.

**Table 1:** Demographic characteristics of youths with IGD and NCs.

Variable	Youths with IGD (*N* = 48)	NCs (*N* = 32)	Statistics	*P* value
Age (years)	20.58 ± 0.96^a^	21.06 ± 2.18	*t* = −1.17	0.25
Education (years)	14.54 ± 0.92	15.00 ± 2.14	*t* = −1.14	0.26
IAT total scores	68.90 ± 8.22	22.72 ± 3.79	*t* = 29.71	<0.01
Reported game playing time (h/week)	23.17 ± 3.64	7.44 ± 3.37	*t* = 19.50	<0.01
Scores of DSM-5 criteria	5.96 ± 1.01	1.88 ± 0.79	*t* = 19.25	<0.01

*^a^Mean ± SD*.

*IAT, Internet addiction test; IGD, Internet gaming disorder; NC, normal control*.

### MRI Data Acquisition

Magnetic resonance imaging scans were obtained by using a 3.0 Tesla Magnetom Trio Tim (Siemens Medical System, Erlangen, Germany) at the Department of Medical Imaging, The Affiliated Wuxi People’s Hospital of Nanjing Medical University. Foam pads were used to reduce head motion and scanner noise. Three-dimensional T1-weighted images were acquired by employing a 3D-MPRAGE sequence with the following parameters: time repetition = 2,300 ms, time echo = 2.98 ms, flip angle = 9°, matrix size = 256 × 256, field of view = 256 mm × 256 mm, 160 sagittal slices, slice thickness = 1.2 mm, acquisition voxel size = 1 mm × 1 mm × 1.2 mm, and total acquisition time = 303 s.

### MRI Data Processing

To identify cortical alternations in youths with IGD, an SBM was performed using the CAT toolbox^2^ with the SPM12 software.^3^ A detailed description of the processing procedure of the CAT toolbox can be found elsewhere.^4^ In brief, this toolbox uses a fully automated method that allows for measurement of CT and reconstructions of the central surface in one step. It uses a tissue segmentation to estimate the white matter (WM) distance, then projects the local maxima (which is equal to the CT) to other gray matter voxels by using a neighbor relationship described by the WM distance. This projection-based thickness allows the handling of partial volume information, sulcal blurring, and sulcal asymmetries with no need of explicit sulcus reconstruction (36). For statistical analysis of surface measure, the CT images were smoothed with a 15 mm full width-half maximum Gaussian kernel.

### Statistical Analysis

To detect statistical significance of group differences in demographic variables between youths with IGD and NCs, the Student’s *t*-test was used. To determine the cortical changes in youths with IGD, we used an analysis of covariance model with diagnostic group as fixed variable, including age as the confounding covariate. Whole-brain peak-level family wise error corrections with *P* < 0.05 (two-tailed) were used in all comparisons to ensure the statistical significance. Then, to further delineate the association between the cortical morphology and symptom severity (reflected by total scores of IAT) in youths with IGD and NCs, respectively, a multiple regression model with IAT total scores as the independent variable was used. Since education level and age were significantly correlated within youths with IGD (*P* < 0.001) and NCs (*P* < 0.001), the multiple regression model included only age as a confounding covariate. For exploratory analysis, we relaxed the peak-level significance threshold to 0.001 (two-tailed, uncorrected) and the cluster-level significance threshold with cluster-size >100. The scatter plot of the relationship between IAT total scores and the mean values of CT was created using GraphPad Prism.^5^ Identification of brain regions was determined with the Desikan–Killiany brain atlas (37).

## Results

Forty-eight youths with IGD and 32 NCs were analyzed in the present study. No significant differences were detected between youths with IGD and NCs in age and education. Compared with NCs, youths with IGD showed a significant increase in IAT total scores and reported game playing time and scores of DSM-5 criteria (Table 1).

In comparison with NCs, brain areas with significantly increased CT were found in youths with IGD, including the bilateral insulae and the right inferior temporal gyrus (Table 2). Moreover, significantly decreased CTs were found in several brain areas in youths with IGD, including the bilateral banks of the superior temporal sulci (STS), the right inferior parietal cortex, the right precuneus, the right precentral gyrus, and the left middle temporal gyrus (Table 2). In youths with IGD, the regression analysis revealed that the CT values in the left insula were positively correlated with IAT total scores (cluster size = 285, peak coordinate MNI*_xyz_* = [−38, −1, −6], *t* = 4.19, see Figures 2A,B). Compared with NCs, youths with IGD showed significantly increased means of the left insular CT (Figure 2C). No significantly negative correlations were observed between the CT and IAT total scores in youths with IGD. In addition, no significant correlations were observed between the CT and IAT total scores in NCs.

**Table 2:** Brain regions showing group differences in CT.

Index	Cluster size	Peak MNI coordinate			Peak *t* value	Side	Overlap of DK atlas^a^ (%)	Region
		*x*	*y*	*z*				
**Youths with IGD > NCs**								
1	1,474	38	−17	1	6.02	Right	100	Insula
2	531	−40	−10	2	5.57	Left	100	Insula
3	33	57	−47	−22	4.68	Right	100	Inferior temporal gyrus

**Youths with IGD < NCs**								
4	136	46	−45	12	−4.93	Right	61	Banks of the superior temporal sulcus
							39	Inferior parietal cortex
5	159	19	−70	31	−4.85	Right	100	Precuneus
6	14	−48	−35	−4	−4.55	Left	54	Banks of the superior temporal sulcus
							46	Middle temporal gyrus
7	7	49	−8	32	−4.53	Right	100	Precentral gyrus

*^a^Desikan–Killiany brain atlas*.

*CT, cortical thickness; IGD, Internet gaming disorder; NCs, normal controls*.

## Discussion

The present study used the SBM approach to characterize cortical morphological features in youths with IGD. The primary finding was that youths with IGD had significant CT alterations in distributed cerebral areas, including the insular, parietal, temporal, and frontal cortices. Particularly, youths with IGD showed a significant association between increased insular CT and IGD symptom severity (reflected by IAT total scores). These findings provide new evidence of cortical morphological abnormalities in IGD and highlight a key role played by the insula in the symptom manifestation of this disorder.

**Figure 1:**
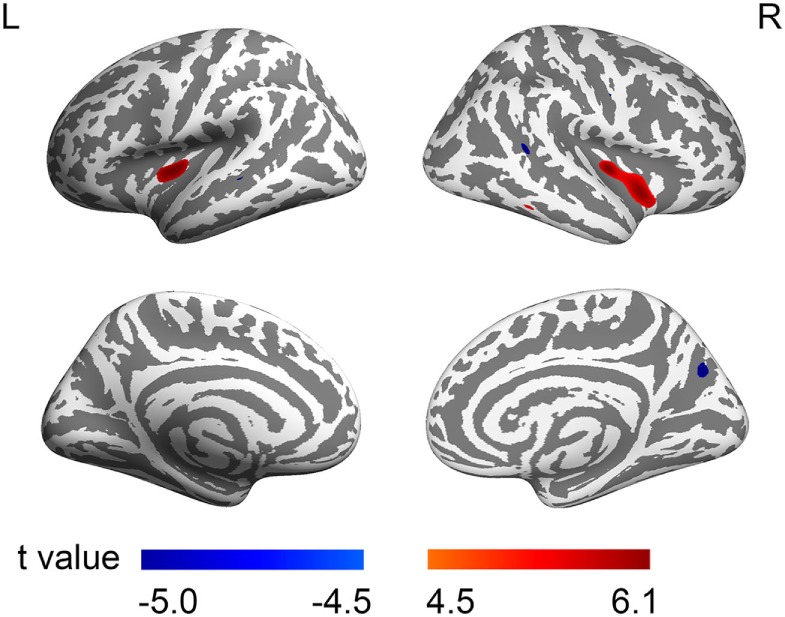
Brain regions with abnormal cortical thickness (CT) in male youths with Internet gaming disorder (IGD). The warm color denotes the brain regions having increased CT, and the cold color denotes the brain regions having decreased CT in youths with IGD. The colored bars show *t* values. L, left; R, right.

Previous studies have demonstrated several specific brain regions associated with IGD, such as the amygdala (38), the insula (39, 40), the precuneus (41), and the middle temporal gyrus (42). In line with the literature, the present study revealed a distributed pattern of CT abnormality in youths with IGD, including the insula, the superior temporal sulcus, the precuneus, the precentral gyrus, and the middle temporal gyrus (Figure 1). Previous studies have shown that Internet game playing was associated with the brain regions responsible for attention and control, impulse control, motor function, emotional regulation, and sensory-motor coordination (14). It is thus conceivable that multiple brain regions have been reported to be probable neural substrates in Internet addictive behavior (38, 39, 43).

**Figure 2:**
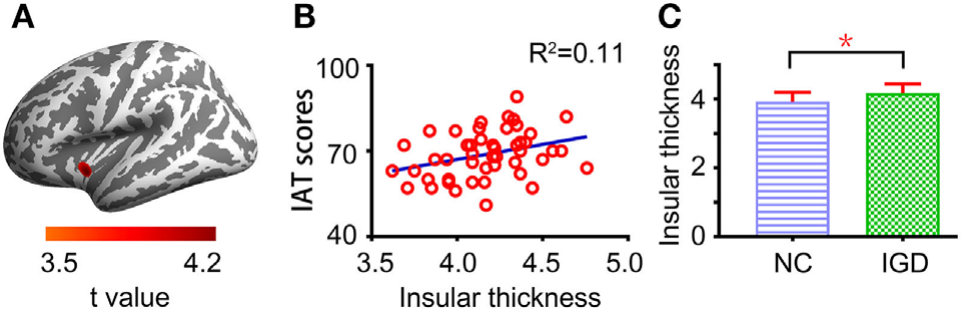
**(A)** The panel shows the left lateral brain image depicting a correlation between the left insular cortical thickness (CT) and Internet Addiction Test (IAT) total scores in male youths with Internet gaming disorder (IGD). The image was peak-level thresholded at *P* < 0.001 (two-tailed, uncorrected) and cluster-level thresholded at cluster size > 100 for exploratory analysis. The warm color denotes the positive correlation, and the colored bar shows *t* values. **(B)** The scatter plot shows the relationship between means of the left insular CT and IAT total scores in youths with IGD. *R*^2^, the coefficient of determination. **(C)** The histogram illustrates group means of the left insular CT for youths with IGD and normal controls (NCs).*P < 0.05. Error bars reflect the SD.

Among our findings, the abnormality of the insular CT and its association with IGD symptom severity were especially interesting. This finding was in line with the previous structural MRI studies (17, 25, 44), which convergently demonstrated structural changes of the insula in individuals with IGD. Our data were also in line with recent functional MRI studies, one of which reported enhanced activity of the bilateral insulae in individuals with problematic Internet use during a monetary incentive learning task (45). Zhang et al. (39) observed an impaired functional connectivity pattern of the insula in subjects with IGD, and their finding was supported by another research which reported an association between IGD severity and insula-based functional connectivity (40). In terms of function, the insula is believed to play a major role in diverse functions such as multimodal sensory processing (46), social decision making (47), emotional experience (48), and motor control (49). Furthermore, the insula is proposed to integrate internal and external information to raise an awareness of the “global emotional moment” experiences that aid in maintaining a context relevant homeostatic state (50, 51). Neuroimaging and lesion studies have suggested that the insula plays an important role in cigarette smoking behavior (52, 53). Moreover, Tanabe et al. reported that the insula cortex was thicker in substance dependent men (26). According to the proposed tripartite neurocognitive model of IGD (15), the insula should be one of the key components underlying IGD, which maintains the craving for an Internet game. The activity of the insula may enhance the drive to play the Internet game and weaken the inhibitive abilities regarding this action. Thus, our study provided new supporting evidence for this model of IGD and highlighted that involvement of the insula in IGD is similar to those of substance addiction. Such information may help to develop effective intervention strategies. For example, psychopharmacological treatments and psychotherapy targeting the circuits including the insula may be effective in weakening craving in individuals with IGD. On the other hand, our results were comparable with the findings derived from a previous independent SBM study, which reported reduced CT of the left insula in individuals (12 males and 6 females) with online gaming addiction (25). Inconsistent with their hemilateral change pattern of CT in the insula (25), the present study showed increased CT of the bilateral insulae in individuals with IGD. One possible reason for the inconsistent results was the differences in sex composition of the samples. Previous studies have revealed that sex is an important modulator of Internet-related behavior (54). Although the effect of sex on the insular CT in individuals with IGD is still unclear, a recent SBM study demonstrated a diagnosis-by-sex interaction on insular CT in substance-dependent individuals (26). Other possible reasons may be related to the methodology, the sample size, and the heterogeneity of participants. The specific roles of insula in IGD require further investigation in future studies by employing a more comprehensive design. Altogether anatomical and functional abnormalities in the insula were widely implicated in IGD. Our findings extend current knowledge about IGD-related insular cortical morphological characteristics and their associations with clinical symptoms.

Another interesting finding of the current study was the significantly decreased CT in the bilateral banks of STS. The banks of STS, defined as the posterior aspect of STS (37), are involved in the processing of various activities such as recognition of motion and faces and understanding of social cues (55). A recent functional MRI study provided evidence that the posterior STS serves as the hub for the distributed brain network for social perception (56). This suggests that the posterior STS is functionally tightly coupled with other brain circuitries and likely integrates social signals processed by more specialized subsystems (56). Furthermore, experimental studies have indicated the role of STS in both real-life situations and games (57, 58). On the one hand, the gray matter density of the STS was specifically associated with online social network size in healthy participants (58). On the other hand, swear words induced more activation in the STS when compared with negative words in young adolescents with IGD (59). A recent meta-analysis also confirmed that STS has been implicated in “the theory of mind” during human–human interactions (60). Therefore, we believe that our finding of involvement of the STS in IGD is a conceivable consequence, which sheds light on the underlying brain structure in IGD. However, the specific roles of the STS in IGD require additional investigation in future studies by employing a more comprehensive design model considering both the structural and functional requirements.

Several issues need to be further considered. First, we employed a recently proposed projection-based thickness approach (36) for measurement of CT in the present study. Such projection-based thickness approach enables the processing of partial volume information, sulcal blurring, and sulcal asymmetries with no need for explicit sulcus reconstruction either *via* skeleton or thinning method and may be superior in certain respects to previous approaches (22, 36). Second, whether these abnormalities observed in our data were a consequence or precondition of IGD remains a question yet to be answered. The answer requires further investigation in future studies by employing a more comprehensive design. Third, the Desikan–Killiany brain atlas handles the insula as a whole region. However, functional MRI and histological studies have shown that the insula is not a homogenous cortical region, which could be functionally subdivided into several distinct subregions (61, 62). Future SBM studies are encouraged to employ an atlas with fine subregional structures of insula. In addition, previous studies have demonstrated that behavioral and neural mechanisms of IGD mostly overlap with those of substance use disorders (18). Thus, more cognitive measurements such as rewards, cravings, and memory-related tasks are needed to explain the findings of the present study.

## Conclusion

Taken together, our data demonstrated that youths with IGD had significant CT alterations in distributed cerebral areas, including the insular, parietal, temporal, and frontal cortices. Particularly, youths with IGD showed a significantly positive correlation between symptom severity and the left insular CT. This work extends current knowledge about IGD-related cortical morphological features and their associations with clinical symptoms. Such information could help with future efforts to identify the role of the insula in the disorder.

## Ethics Statement

This study was carried out in accordance with the recommendations of the Medical Ethics Committee of The Affiliated Wuxi Mental Health Center of Nanjing Medical University with written informed consent from all subjects. All subjects gave written informed consent in accordance with the Declaration of Helsinki. The protocol was approved by the Medical Ethics Committee of The Affiliated Wuxi Mental Health Center of Nanjing Medical University.

## Author Contributions

ZZ and LT designed the study. FZ, JL, QT, JW, and LC contributed to the acquisition of the data. LT, SW, and JL analyzed the data, interpreted the results, and drafted the manuscript. All the authors critically reviewed content and approved the final version for publication.

## Conflict of Interest Statement

The authors declare that the research was conducted in the absence of any commercial or financial relationships that could be construed as a potential conflict of interest.

## Footnotes

^1^http://www.cnnic.net.cn (Accessed: March 17, 2018).

^2^http://dbm.neuro.uni-jena.de/cat/ (Accessed: March 17, 2018).

^3^http://www.fil.ion.ucl.ac.uk/spm/software/spm12/ (Accessed: March 17, 2018).

^4^http://dbm.neuro.uni-jena.de/cat12/CAT12-Manual.pdf (Accessed: March 17, 2018).

^5^https://www.graphpad.com (Accessed: March 17, 2018).
